# Framing heuristics in inclusive education: The case of Uganda’s preservice teacher education programme

**DOI:** 10.4102/ajod.v8i0.611

**Published:** 2019-10-21

**Authors:** Proscovia S. Nantongo

**Affiliations:** 1 Department of Education, Faculty of Educational Sciences, University of Oslo, Oslo, Norway; 2 Department of Special Needs Studies, Faculty of Special Needs and Rehabilitation, Kyambogo University, Kampala, Uganda

**Keywords:** inclusive education, framing analysis, socio-historical factors, narrow framing heuristic, teacher training

## Abstract

**Background:**

Recent education-related research has raised concerns about the persistent exclusion of vulnerable learners in Uganda. The Revised Primary Teacher Education Curriculum of 2013 marked an ambitious yet inconclusive attempt to advance the implementation of inclusive education but has encountered deeply entrenched sociocultural exclusionary practices among education experts.

**Objectives:**

This study aimed to explicate education practitioners’ interpretations of Uganda’s flagship inclusive education programme in preservice primary teacher education.

**Method:**

Drawing on the conceptual vocabulary of frame analysis and the qualitative analysis of individual and group interviews and classroom observations, the interpretations of inclusive education implementation in preservice primary teacher education in Uganda were examined. The participants included policy design experts, curriculum design experts and classroom practitioners.

**Results:**

Three main findings emerged. Firstly, interpretations of inclusive education displayed a narrow framing heuristic of inclusive education as a perfunctory, daily practice rather than a pathway for reflective, inclusive pedagogical engagement. Secondly, the heuristic encouraged the treatment of inclusive pedagogy as a ‘label’ under a specific rubric referring to sensory impairments or disabilities – a historical device for sociocultural exclusion. Thirdly, inclusive education was a praxis but was misframed from its original intentions, causing tension and resentment among practitioners. These findings contribute to the debates on the sustainability of inclusive education beyond preservice teacher education.

**Conclusion:**

Uganda’s flagship inclusive education programme in preservice primary teacher education was fraught with tensions, ambiguities and an overt, urgent need for change.

## Introduction

In most sub-Saharan African countries, and in Uganda in particular, numerous studies on global education reforms have revealed contradictory implementation strategies to achieve the set goals (Charema 2010; Hardman et al. 2011; Munene 2016; Shevlin & Kearns 2010; Zajda 2018). Nketsia, Saloviita and Gyimah (2016) and Slee (2013) have particularly raised concerns on the prevalence of knowledge uncertainties about best practices, leading to a state of trial and error among inclusive reform implementers (i.e. teacher educators). What is at stake therefore is the sustainability of the reform implementation as prioritised by the United Nations Educational, Scientific and Cultural Organization (UNESCO) (2014). In Uganda, education reform, geared for all school-eligible learners, has failed the majority of the once-enthusiastic vulnerable population (Munene 2016). Instead, they continue to be socially victimised, making them prey to the same reforms once intended to offer alternatives. For instance, Slee (2013) argues that exclusively designing specialised teacher training programmes and establishing special grants for categorised learners are a myopic interpretation of the intent of inclusive education and directly contribute to exclusion. Amidst such contradictions, O’Sullivan (2005) calls for research-based solutions to failing reforms.

Since 2013, Uganda’s preservice teacher education programme has undergone a substantial reform process. For example, as the product of the reform, the present teacher education curriculum includes two course subjects – special needs education (SNE) or inclusive education and information, communication and technology (ICT) – among other changes. This reform process is in line with the UNESCO’s^1^ aim to establish ‘institution-wide approaches to Education [for] Sustainable Development (ESD) at all levels’ and ‘to jointly develop a vision and a plan to implement it’ (UNESCO 2014:35). Uganda’s focus on broadening the existing Grade III Teachers Certificate in the Primary Teacher Education Programme by including special needs/inclusive education was a social change towards social justice for all (Gallagher 2006; Shevlin & Kearns 2010; Slee 2013; Unianu 2012). However, the curriculum reform effort had no specific policy streamlining the activities unlike other educational practices, such as universal primary education. In this context, inclusive education implementation in Uganda partially overrules the argument that ‘policy development and philosophical thought outpace practice’ (Hodkinson 2010:61).

Further research on global education reforms reveals that individual states cherish different sociocultural values perhaps because of their historical backgrounds. And as Hargreaves and Shirley (2009) assert, there is a direct relation between those values and the extent to which the education reforms are achieved. In other words, the states’ multi-faceted definitions of given reforms determine the unique ways of implementation (Zajda 2018). Moreover, individual and social perceptions of such reforms create a sort of social values continuum, which, in turn, becomes a social determinant for reform implementation (Coleridge 1993; Slee 2013; Wertsch 1979). We question to what extent a particular reform conforms to the social goal at present. The present study, therefore, aimed to explore how Uganda’s revised Grade III teacher education curriculum addresses the understanding of inclusive education interpretation. Specifically, in 2015 and 2017, a convenience sampling was performed to recruit participants from five public institutions representing three levels: (1) policy design experts (macro-level), (2) curriculum design experts (meso-level) and (3) classroom practitioners in teacher training (micro-level).

The structure of this article is as follows: firstly, the article describes discourses on inclusive education strategies in Uganda. Secondly, it presents how framing a theoretical concept and framework as proposed by Goffman (1974) addresses the participants’ interpretations of inclusive education. Then, it outlines the research methodology, followed by a presentation on the results. Next, the three main findings are discussed through the lens of framing theory. Finally, the article highlights concerns for future research on the sustainability of inclusive education and teacher education.

### Context of inclusive education in Uganda

Inclusive education is a priority area identified by the Higher Education and Multimedia in Special Needs Education and Rehabilitation (ENABLE), a partnership project aimed at developing teacher competence at higher institutions of learning in Uganda, Kenya and Tanzania.

Inclusive education has developed considerably since Uganda achieved its independence in 1962, with efforts concentrated on addressing and amending several types of distributive injustice, including education and economic inequalities (Munene 2016). Despite some individualised educational achievements in terms of ethnicity and gender, nearly six decades after independence, Uganda Bureau of Statistics (2017) reports that the economic gap between the poor and the rich steadily widens. In terms of gender parity, since the 1990s, steady progress has been reported on primary education enrolment, completion rates and academic performance assessments (Ministry of Education and Sports [MoES] 2016). Moreover, such progress has not been replicated or reported among learners with special needs (Ojok & Wormnaes 2013; Riche & Aniyamuzaala 2014). As Coleridge (1993) stresses, the close relationship between poverty and impairments exacerbates certain sociocultural exclusion practices.

The colonial and post-colonial eras provided institutionalised special education for persons with disabilities in specified schools and homes. Persons with impairments were hitherto believed to be socioculturally uneducable and were thus excluded from formal education (Karugu 1988). However, persistent worldwide agitation for access to formal quality education for all school-eligible learners, in a socially healthy environment, led to the enrolment of high numbers^2^ of previously segregated and excluded learners in regular schools^3^ in the case of sub-Saharan states (Munene 2016). However, UNESCO (2014) report reveals emerging setbacks in learner enrolment, retention and education quality. As O’Sullivan (2005) recommends, the report highlights an urgent need for policy research to support practical solutions to educational exclusion.

Scholarly works on educational reforms report contradictory understandings and interpretations of reform processes at different levels (Elton 1979; Terzi 2014; UNICEF 2015). In the chapter, ‘Education can change society?’, Elton (1979:72) advances the argument that ‘successful change is a result of the social value attached to it’. According to Torre (2017), specific social environments attract particular responses from specific participants. Elton (1979) and Torre (2017) both shed light on the interplay between global inclusive education discourses and Ugandan sociocultural perceptions of inclusive education amidst diversities. Based on the inadequate interpretations and implementation of earlier educational reforms (Altinyelken 2010; Charema 2010; Munene 2016), the researcher assumes that there is knowledge uncertainty among educational practitioners about inclusive education reform as has been the case with others. In fact, according to Tschannen-Moran and Chen (2014), inadequacies in education reform initiatives arise from mismanagement.

Uganda’s action, in 2013, to launch a systematic, national-scale preservice primary teacher training initiative by revising the existing preservice teacher training curriculum to include SNE^4^ course content addressed the global demand for rethinking inclusive education (UNESCO 2014). The aim of the present study was to contribute to the understanding of how this nationwide programme, which addresses inclusive education for preservice teacher preparation, has been implemented. Meanwhile, a full-fledged distance learning programme – leading to a diploma in SNE – has been in existence for some years under the ownership of Kyambogo University. Inclusive education reform discourses operate on three levels: macro-level, which is the political or macro-managerial wing; meso-level, which is the expert or supervisory wing; and micro-level, which is the field operational wing. These levels are the subject of this study because of their intertwined roles as mentioned in the methodology section. And in that view, their mutual interpretation of the education reform leads to achieving the reform goals.

### Conceptual vocabulary of framing analysis

Bateman (1976) and Goffman (1974) pioneered the research on framing. This concept has had a significant influence on both conceptual and methodological analyses of present social problems (Benford 2010; Benford & Snow 2000; Dalkilic & Vadeboncoeur 2016; Davies 1979; Hetland 1996; Johansson 2007). Numerous research traditions in the field of framing analysis are grounded in Goffman’s (1974) fundamental question, ‘what is it that’s going on here?’ – a question that draws attention to ‘an individual’s particular point of view’ (Goffman 1997:226). An individual’s interpretation of the experience in a given encounter constitutes his or her perceived reality (Goffman 1974; Johansson 2007). Moreover, no individual has the capacity to establish any sort of absolute reality. Rather, the individual must be able to locate, perceive, identify and label his or her experiences in relation to the sociocultural context (Benford 2010; Benford & Snow 2000; Goffman 1997). In doing so, the individual frames his or her experiences accordingly.

#### The sociocultural contextual framing of inclusive education

Using the lenses of Goffman (1974) to frame the understanding of the implementation of educational reforms, we observe what Hetland (1996) describes as double framing within which he cites the possibility of conflicts. For example, he urges that ‘frames are both inclusive and exclusive’ (Hetland (1996:15). The tendency to include while framing, may be framed by others as excluding. In other words, what may be experienced by framing is a diversion from the intended social goal – such as inclusion for all. These hiccups, or what Bacchi (2000) describes as misframing, occur:

[*N*]ot only because reform efforts are opposed, but because the ways in which issues get represented have a number of effects that limit the impact of reform gestures. [I]ssues get represented in ways that mystify power relations and often create individuals responsible for their own ‘failures’, drawing attention away from the structures that create unequal outcomes. (p. 46)

Therefore, to explore the implementation of inclusive education, we must seek meanings in the socially shared goals within the sociocultural context, as alluded to by Lev Vygotsky (Wertsch 1979), and expanded on by his proponents (such as Cole & Engeström 1993; Cole & Gajdamaschko 2007; Daniel 2009). And as a social quality, Voogt, Pieters and Handelzalts (2016) suggest that collaborative framing in inclusive education (global reforms) leads to success in the stakeholders’ implementation process. Similarly, Davies (1979) and Bacchi (2000) argue that the real implementation process lies within social limitations. For example, language, as a fundamental social resource, frames individuals’ perceptions within social networks. Language has an enormous influence on framing cultural practices, such as attitudes and actions (Coleridge 1993; Torre 2017; Wertsch 1979). For instance, Coleridge (1993:100–101) cites an inconclusive debate on languaging (framing) as in the phrases, ‘people with disabilities’ or ‘disabled’ or ‘impaired’ people. But there is no doubt, that social conceptions and perception ultimately influence the extent of social inclusion (Coleridge 1993) such as formal education. Relating inclusive education to destitute learners prejudices the quality of education.

The conceptual vocabulary of framing analysis, therefore, guides this study’s understanding of participants’ interpretations of inclusive education implementation at three interrelated levels: macro, meso and micro. The assumption was that successful implementation of inclusive education, like other global education reforms, largely depends on sociocultural realities.

## Study design and methodology

This study was based on qualitative data collected in two phases, in 2015 and 2017, from five key institutions in primary teacher education in Uganda. One institution had authority over policymaking, one was responsible for teacher curriculum development and three were primary teachers’ colleges (PTC) involved in classroom teaching. Uganda has 45 public PTCs with more or less shared socio-historical characteristics (Hardman et al. 2011) such as founding bodies, same recruiting process, same curriculum, among others. This study is a part of the ENABLE partnership project, which aims to develop teacher competence for inclusive education at higher institutions of learning.

### Selection criteria for participants in phases I and II

The three PTCs were conveniently sampled based on the researcher’s experiences working with them in previous national academic engagements. This prior knowledge enabled the completion of the data collection process within the desired timeframe. Otherwise, other PTCs could have provided reliable data (Fraser & Bedford 2008).

In phase I, one of the ENABLE project managers contacted the managers of the five institutions by email and telephone, relaying our intentions to study inclusive education implementation from the perspective of preservice teacher education. With this background, these managers mobilised and recruited all of the study participants. A total of 16 participants (4 men and 12 women) from four institutions attended group interviews. The group sizes ranged between three and five participants, while the interview durations ranged between 45 min and 1 h and 25 min. The variations occurred during the interview process. The fifth institution offered one participant for an individual interview. In this phase, we aimed to strengthen the ENABLE partnership by accessing reliable information from valid sources so as to establish a research rationale for this study as emphasised by McDermott, Gospodinoff and Aron (1978).

In phase II, I conveniently identified 12 individual interviewees, including nine teacher educators (four men and five women) from the three PTCs that participated in phase I. Using a list of teacher educators provided by the PTCs, I contacted potential participants by telephone and sought their consent to participate. This was the most convenient means at the time, given the ongoing activities of the PTCs. As a result, three out of 12 participants also attended phase I. In addition, three in-service teacher trainees (one man with experience teaching a child with cerebral palsy and two women with sensory impairments) were purposively recruited based on their personal experiences with the Grade III teacher training programme. In addition, they were at the time among the in-service teacher trainees attending the SNE or inclusive education programme which the PTC teacher educators facilitated. Four lessons were also observed. The duration of the interviews and the class observations varied.

Complying with ethical research standards, all recruited participants gave informed consent, and their contextual anonymity is preserve in this article by using only the institutional levels.

### Data generation procedure

In phase I (2015), the research interest focused on the implementation of inclusive education in teacher training. In phase II (2017), the data and experience acquired in phase I facilitated an in-depth study of the participants’ interpretations. The data were generated from three main sources: (1) four group interviews, including one group with three persons from a policy institution (macro-level) and three groups from PTCs with a total of 11 persons (micro-level); (2) 13 individual interviews, with one curriculum developer (meso-level), nine teacher educators and three student teachers (micro-level); and (3) four classroom observations (micro-level).

During the interview process, I deliberately attempted to stress the inclusive education discourses in the teacher training programmes by asking open-ended questions and using probing techniques. From the ongoing data analysis, it was revealed that, in phase I, some individuals’ contributions dominated the group sessions, while others exhibited passive tendencies, which were displayed as general consensus. In phase II, I took a different approach, focusing on individual interviews to document personal experiences (Merrill & West 2009) based on reality (Huang & Carspecken 2013; Jørgensen & Phillips 2002) while limiting hearsay utterances. In addition, the use of ambiguous prompts, such as ‘I also want to take this opportunity to request that you tell me about yourself, if you don’t mind’ enhanced the respondents’ feelings of liberty. As in other scholarly works (Beynon & Dossa 2003; Dautenhahn 2002; Maynes, Pierce & Lasletts 2008), the participants’ narratives potentially enabled access to self-understandings and critical interpretations of individualised experiences.

The data generation was not built on standardised questions, but rather on a flexible technique. Firstly, the researcher observed classroom teaching facilitated by the same teacher educators participating in the study. Then the researcher presented the general interests of the study, permitting the participants to self-identify. The interview process unfolded based on what had already been shared, for example knowledge regarding inclusive education from observed lessons, previous trainings, implementation possibilities for inclusive education in teacher training and ICT trainings, stakeholders’ willingness/readiness to promote inclusive education in colleges, general teacher education pedagogical strategies for inclusivity and possible recommendations for effective implementation of inclusive education reform. In brief, the interview process was backed by the UNESCO (2014) roadmap for implementing the revised curriculum and the policy discourses on staff recruitment. The interviews were audio-recorded and the classroom observations were video-recorded to preserve the original messages.

The researcher coded the data along the subject of inclusive education reform and teacher education. Samples of the coded data from the interviews were presented to the research group of which the researcher was a member for critical analysis.

Despite the perceived bias of insider research (Taylor 2011), my prior knowledge of the field created a synergy between what was visible and what was relative to the socio-historical practices, which Fischer (2017) recognises as a strength of qualitative research – for example, asking the right question with the right tone to elicit a genuine response.

### Framing analysis of inclusive education practices

Framing analysis was used to understand how the participants interpreted the implementation of inclusive education in the preservice teacher training programme. NVivo software was used to transcribe the data from the recorded interviews and the classroom observations. Multiple readings of the transcribed data generated categories or frames corresponding to the study aim of exploring education practitioners’ interpretation of inclusive education in primary teacher education, and scholarly insights on framing formed the study’s analytical strategy. Further analysis of findings by Per Hetland (1996:15) revealed four ways in which ‘double framing’ can emerge: ‘conflicting, competing, incompatible or compatible framing’. Similarly, there is a strong assumption that educational practitioners interpret the implementation of inclusive education reform within particular frames.

### Ethical consideration

All possible ethical measures with respect to data generation, storage, processing and reporting were detailed and submitted to the authorities beforehand. In response, the author received a clearance certificate (Ethical clearance number: NSD 53561). The author has not deviated from the position unless advised.

## Presentation of the findings

The frame analysis identified two themes: (1) the conceptualisation of inclusive education and (2) the empirical interpretation of the implementation of inclusive education in the teacher training programme.

### Conceptualising inclusive education in preservice primary teacher education

Most participants in both phases, and at all three levels, used the concepts of SNE and inclusive education interchangeably as a matter of convenience in the present study. However, SNE directly refers to teaching children with impairments or disabilities, and inclusive education refers to the physical presence of learners with impairments alongside learners without impairments. Throughout the study, there was only one instance when a teacher educator referenced poor and orphaned families (vulnerable learners) as beneficiaries of inclusive education. The following submission from a meso-level participant clarifies on the practical interpretation of both concepts:

‘If [*the impairment*] is out of order, then you must recommend to—we have specialised schools in case a person cannot hear completely. Are you following me? But there are these special needs for which, in a class, you can afford inclusion. A person has a leg injury; I mean, a leg disability, so he is able to hear, listen and write and keep up with others. So we teach [*teachers*] to manage that.’ (curriculum expert, 2015)

Some participants cited related conceptual misunderstandings of inclusive education among stakeholders. Some micro-level participants shied away from inclusive education because they perceived it as pedagogically inferior and simply a convenient policy tool. At all levels, the participants revealed a mismatch among various stakeholders’ interpretations, largely influenced by deeply seated negative mindsets towards academic competence of impaired children. An example from an interviewee with sensory impairment underscores the importance of social attitudinal change. She quoted a previous encounter with a PTC staff member, who said, ‘For us here, we don’t train blind people. How [*could*] we handle you? We do not know how to train blind people!’ (director of studies in charge of teacher training) The interviewee concluded that such attitudes directly undermine the implementation of inclusive education at all levels. This view was common in participants at all levels.

The Ugandan government’s conceptual interpretation of inclusive education at teacher training level focused on social inclusivity through the construction of a model PTC with ramps and wide doors to promote physical accessibility. As one meso-level participant put it, ‘[*y*]es, let’s allow them in class. Let’s teach them [*and*] use materials that are locally available and not harmful’ (teacher educator with SNE exposure). This modelling strategy of inclusive education could be replicated by policy designers and infrastructure developers.

In addition, the identification and categorisation of learners (with impairments) were emphasised in teacher trainings to guide their choice of teaching approaches ‘[*b*]ecause some … teachers cannot tell that the child has this kind of challenge’ (micro-level group participant, 2015) and provide the appropriate support required, such as the ideal classroom seating arrangements for learners with hearing impairments (micro-level participant 2017). The revised curriculum also included basic sign language and braille trainings, and strong emphasis was placed on assessing learners’ abilities to cope with regular classroom norms.

### Frames of inclusive education implementation in primary teacher education

The second theme considered how the participants perceived the implementation of inclusive education in the teacher training programme, and the findings were consistent with my initial assumption that inclusive education relies on a multi-level approach. At the policy level, the renaming of the Department of Special Needs and Inclusive Education in the MoES was purposively done to prioritise both SNE and inclusive education in policy discourses. As described by the curriculum experts who participated in this study (meso-level, 2015), both special and inclusive schools exist; the former are primarily meant for SNE learners, while the latter are ordinary or regular or normal schools that admit children with special needs. One participant emphasised that the minimal requirements for inclusion are the ability to hear, write and, most importantly, keep up with others in normal schools.

The findings revealed progressive efforts to enact a comprehensive policy provision to regulate inclusive education practices in Uganda. In fact, during policy consultations, several stakeholders pledged to support inclusive education. One macro-level group participant noted, ‘[*i*]t is the way to go’ (policymaker, 2015). Further findings revealed that the ministry was negotiating international and domestic partnerships to support SNE or inclusive education implementation among teachers in Uganda and the region. External support has spearheaded previous initiatives in the provision of SNE since Uganda gained independence in 1962 (Karugu 1988).

Although there was consensus that the revised curriculum can effect inclusion, most interviewees expressed shared doubt on the content, framing the curriculum as insignificant and inadequate to prepare the teachers to teach learners with sensory impairments. The curriculum implementation process was under-resourced. According to the curriculum expert, the revised curriculum did not target learners with severe impairments; rather, such learners would be recommended for special schools with specialised teachers. He held the opinion that, instead of overloading the current curriculum, a separate preservice teacher training programme on SNE should be established. The threat to the curriculum was also raised by a macro-level group participant:

‘Everybody has said [*it*] very many times. I don’t know whether [*overloading the curricula has ceased*] or not. It is this [*role of the*] research [*findings*] again to tell us.’ (policymaker, 2015)

At the practical level, one hour of teaching per week was allocated to SNE or inclusive education to equip preservice teachers with pedagogical skills. As a practice, teacher educators are specialists in particular subjects, which they teach at the PTCs. The practice is the same for SNE. However, at the start of the revised programme in 2013, only four out of 45 PTCs had recruited SNE teacher educators. Since then to date, the process was halted because of financial implications at the macro-level. In fact, the interviewees confirmed that all 45 PTCs lacked SNE teacher educators, although a few individual PTCs had privately arranged for SNE teaching. Furthermore, it was clear from the classroom observations that the SNE content was focused on passing the national examinations. Furthermore, the PTCs incurred unbearable financial costs by hiring external teaching manpower in order to sustain SNE implementation. These challenges were acknowledged by participants at all three levels.

In-depth probing on why this situation could not be challenged revealed resentment. Representing many voices, a macro-level participant regretted the lack of a comprehensive policy that would address implementation from the policy viewpoint. Meanwhile, a meso-level participant (2015) declined the role of persuing policy-makers to perform their duly designated duties. Rather, he preferred to stick to his role.

The revised curriculum added ICT as a subject; as such, every PTC in Uganda must establish ICT facilities to enable teaching and learning – an integration strategy towards education digitalisation. One of the SNE teacher educators commended the ICT contributions to what she described as a ‘shallow syllabus, coupled with limited access to relevant textbooks’. By downloading materials from various learning sites, she was able to give relevant resources to the students. However, using ICT at the PTCs was seldom and isolated; the PTCs could not financially sustain open ICT access because of large volumes of internet bundles and other technical repairs, leading to inevitable restrictions. For example:

‘Where I have been [*at other PTCs*] and where I am [*now*] and with [*the*] experience which I get from other [*teacher educators*] from other colleges, I and those other teacher educators, we do not use ICT for teaching. I have only observed the ICT person teach computers as [*a*] kind of demonstration. That’s all, but not with these other subjects of the curriculum’ (teacher educator, 2017).

The findings from the three levels revealed appalling conditions, including teacher educators’ pedagogical knowledge uncertainty, insufficient logistics, overloaded curriculum content, competitive academic demands, a lack of specialised teachers and a high teacher educators to student ratio. Furthermore, most teacher educators were reluctant to implement inclusive education citing compromising working conditions. According to the interviews and the revised curriculum, the preservice teachers training programme had two obligatory teaching practice sessions (i.e. 2 weeks of preparation at a PTC and 4 weeks of teaching). A unified voice from all participants at all three levels conceded that within such a short time frame, coupled with examination-oriented performance, inclusive education could not be sustained. On the issue of teacher educators’ SNE competences, the meso-level participant reaffirmed that teacher educators had acquired impeccable skills and were adequately prepared to teach preservice teachers. The findings revealed that the curriculum reform programme was never intended for ‘sophisticated skills’ (2015).

## Discussion

This section discusses the three frames intertwined by the two interrelated themes identified in the findings. Firstly, inclusive education practices were subject to a heuristically narrow framing in which the participants conceived of their interpretation as belonging to the broader sphere of actionable implementation but did not always realise or fully articulate their efforts. In other words, inclusive education intentions and actions were imperfectly actualised and separated by a wide divide. Secondly, related to the narrow heuristic framing was the participants’ tendency to express reality in normative terms (how things should be) and not in descriptive terms (how things are), creating a sense of complacency and idleness. Thirdly, as a direct corollary to the first two frames, the normative manifestations of the concept of inclusive education, as articulated in the classroom observations and the interviews, were fraught with tensions, ambiguities and, simultaneously, an urgent need for change.

### Narrow heuristic framing of inclusive education

A prime finding at all levels was the framing of inclusive education as a concept associated with disabilities or impairments and sometimes destitution. Coleridge (1993) describes this tendency as labelling. Increasingly, critical scholars have been contesting the role of labelling, pointing out its detrimental consequences: ‘labels disable because they focus on the person not as a person but as a case or an object’ (Coleridge 1993:99). In contrast, proponents were more concerned with its ability to identify, mobilise and direct attention to appropriate interventions (Coleridge 1993; Torre 2017). With no intention to take sides, sociocultural scholars contend that the meanings of concepts emerge within specific engagement (Coleridge 1993; Hutchison 1995; Rogoff 2003). From this understanding, we can conclude that inclusive education viewed from the global perspective ignores the cultural power of labelling for actual implementation, which is why this narrows down to disabilities or impairments.

The revised curriculum made inclusive education as an option for learners with impairments, preparing teachers by focusing on skills, such as sign language and braille, and addressing physical accessibility, such as with the construction of ramps. Furthermore, the teacher training programme emphasised identification and assessment with the aim of ‘sorting’ learners. For example, deaf and blind learners were less favoured for inclusion than learners with physical disabilities. The framing of inclusive education within disability or impairments had a sociocultural potential to restrict the range of expectations (UNESCO 2014) and pedagogical decisions (Coleridge 1993).

Further evidences of conceptual perceptions of inclusive education across the three levels suggested a double frame. Hetland (1996) discussed the double framing of a multi-level conflict when one issue is favourably presented but shortly results in contrasting outcomes. Despite the goal of inclusive education to empower every member of society to contribute to social justice and sustainable social development (UNESCO 2014), prevailing disabling implementation conditions, such as the failure to appoint SNE teacher educators to operationalise the revised curriculum, contradicted the government’s commitment to inclusion. The current implementation struggles arose from the precarious practice of confining inclusive education within a specific rubric of SNE without thorough scrutiny of its practical consequences. The lack of specialised personnel and the lack of inclusive pedagogical training of general educators widened the gap among subjects and promoted individual teaching among teacher educators.

The systematic promotion of a divide between SNE or inclusive education and other subjects and teacher educators is of great concern. As Coleridge (1993) observes, negative social attitudes develop with separate programmes, hampering most implementation efforts for persons with disabilities. The allocation of SNE or inclusive education, as a subject, to specialised teacher educators with knowledge of impairments or disabilities limited other teacher educators from trying inclusive pedagogical strategies in their subjects, discouraging them from conducting critical pedagogical analysis to potentially promote inclusion (Bullough, Jr. 2010; Slee 2013; Veck 2014). Slee (2013) observed that further exclusion becomes imminent when available social resources are simply ignored and demobilised. Therefore, based on the findings, I have concluded that the curriculum revision discourses did not directly translate into practice (Hardy & Woodcock 2015), at least not when the interpretation was restricted to impairments (Coleridge 1993; Hardy & Woodcock 2015).

### Tendency to express reality in normative terms

Uganda has stood tall in the region for formulating policies to guide the local implementation of global agendas (Munene 2016; UNESCO 2014). However, the mobilisation of necessary resources to effectively implement such agendas is still contentious. The mere (at times, vague) presence of policies does not guarantee effective implementation (Bacchi 2000; Corbett 2001; Hardy & Woodcock 2015; Hodkinson 2010). In the present study, there is an implied impression that inclusive education discourses were unanimously supported by macro-, meso- and micro-level participants; this positive gesture was demonstrated by the launching of the revised curriculum. These normative responses were pronounced at the group level but tended to disappear during individual interviews in which most participants expressed concerns about inadequate institutional support and additional workloads. Specific concerns included understaffing, inadequate instructional materials, shallow curriculum content and limited knowledge of inclusive education.

Although the study found incidences of SNE teachings and a nationwide strategy to digitalise teaching and learning by establishing ICT centres at every PTC, most micro-level participants, as well as the researcher’s observations, indicated a double framing of reality. Such documented revelations raised concerns about whether the revised curriculum actually transformed pedagogical practices in the teacher training programme. It remained debatable whether the multi-level approach to curriculum reform achieved the intended outcome set by the inclusive education reform.

Normative responses about inclusive education were more common in the group interviews and among the macro- and meso-level participants, but less common among individual interviewees and the micro-level participants. This state of conflict indicates a sociocultural strategy to avoid conflicts that may culminate in job-related consequences. Increasingly, educational reforms are highly politicised and may lead to consequences, such as personal disagreements among implementers (Munene 2016).

### Tensions, ambiguities and a simultaneous, urgent need for change

Overall, the study findings present a potential challenge to the sustainability of inclusive education (UNESCO 2014) in Uganda (Altinyelken 2010; Gallagher 2006; Hardman et al. 2011). The findings display high possibilities for tension, with a push towards the idea that ‘inclusive education is the way to go’ (macro-level group participant, 2015) regardless of the potentially inadequate capacities of the implementers. This tension stems, in part, from ambiguities in the conceptual understanding of the inclusive education reform discourses. As Hetland (1996:83) acknowledged, in the process of translating policy discourses, we encounter concept ‘ambiguity, inconsistency or out spoken disagreements’. And therefore, inclusive education is no exception.

The perceived internationalisation of the UNESCO reforms indirectly compels member states to adopt a normative tendency in addressing social justice. It is not a historical coincidence, therefore, that the implementation of inclusive education operates, in most cases, on external finances and technical support (Hardman et al. 2011; Munene 2016). In stronger terms, the sustainable implementation of inclusive education relies more on a state’s policy rhetoric than on actionable commitment. As Zajda (2018) observes, policy discourses, especially those emanating from global rhetoric, provide little room for critique. Rather, the would-be implementers, such as teacher educators, neither reject nor suggest possible solutions to implementation challenges.

More often than not, policymakers inadequately consult implementers for their field-based views (Bacchi 2000; Shevlin & Kearns 2010). Therefore, a sense of resentment and varying degrees of frustration are expressed; as a meso-level participant submits: ‘I strongly insist [*dealing with implementation challenges*] is not [*in*] my control. It is not my business, and I do not promise to [*intervene*]’ (curriculum expert, 2015). The kind of attitude exhibited in the above resignation can be equated with ‘*to whom it may concern*’, and was common with other participants. No surprise therefore, that in general many PTCs partially implement the curriculum reform. Further still, the findings corroborate Terzi (2014)’s study finding in the sense that there was limited evidence that the teacher educators were pedagogically competent for inclusive education, causing knowledge uncertainty that is likely to promote resistance to attitude change among practitioners.

Hardy and Woodcock (2015) and Unianu (2012) assert that attitudes and perceptions strongly influence individuals’ daily practices. In the case of Uganda, the findings present inclusive education as intended for not only learners with impairments, but also learners hamstrung by poverty. Considering the would-be financial burden on a guardian for educating (Munene 2016) a disabled child, any state attempt for free inclusivity is a great achievement, although we contend that inclusive education goes beyond accessing physical environments to considering inclusive pedagogies among teachers (Dalkilic & Vadeboncoeur 2016; UNESCO 2014). The integration of ICT infrastructure at PTCs, if effectively implemented, would likely contribute to knowledge building, strengthen cooperation among teacher educators and develop partnerships to enhance inclusive education in teacher education.

In the context of Uganda, as remarked by the policymaker, ‘inclusive education is the way to go’. But according to most participants, insufficient implementation of inclusive education was blamed on what Bacchi (2000) deliberates on as the poor packaging of reforms. Indeed, other studies recognise teachers as the effective agents of educational change (Altinyelken 2010; Florian & Linklater 2010; Gallagher 2006) but the lack of institutional support to address the implementation challenges mentioned above has a direct effect on the reform sustainability. One micro-level participant (2015) challenges the state in ensuring that staff recruitment is urgently completed. Otherwise, current misconceptions of inclusive education reform seem to promote affirmative exclusion by simply allowing vulnerable learners physical space with the regular schools, while at the same time limiting their fully pedagogical participation for whole human development.

From the findings, we can conclude that the understanding of inclusive education implementation among education practitioners lacks consistency and serves as a convenient strategy to circumvent unpleasant consequences – because reform experts say that inclusion is the way to go. However, most interviewees across the three levels problematised inclusive education implementation as a complex socio-economic commitment to change that would most likely create tension among implementers (especially with the lack of inclusive pedagogical competencies). Pending demands by teacher educators ranged from institutional and technical support to matters of personal well-being. In my view, the present inclusive education initiatives simply confirm what Munene (2016) observes to be efforts that earn international recognition but still leave much to be desired. In such a case, Zajda (2018:3) appeals to ‘logic and common sense when addressing agendas constructed within a global perspective’. In other words, inclusive education reform implementation should commence from a local socio-historical viewpoint, considering the ‘limitations as well as the strengths’ (Hargreaves & Shirley 2009:147) and avoiding unnecessary heuristics in practice. After all, good policy discourses remain in theory until they are operationalised within a given context (Hardy & Woodcock 2015).

## Conclusion

The three main frames discussed in this study support the following conclusions: (1) inclusive education remains synonymous with special (needs) education and is largely perceived as designed for learners who have impairments and disabilities or/and live in poverty; (2) labelling and categorising learners, teachers and systems significantly affects the daily practices of inclusive education; and (3) the teacher training programme under the revised curriculum fails to meaningfully address inclusive pedagogy – a potential misframing of the inclusive reform intentions. The desired ability to mobilise social resources, generate constructive criticism and build consensus on critical knowledge for implementation is neutralised by the misconceived institutional pedagogical support in preservice teacher education.

### Study limitations

This study presented and discussed findings generated in September 2015 and January 2017 after the curriculum revision in 2013. The time difference between phases I and II of data collection may have had in some way affected the validity of the study conclusions as it is the same as the time difference between the final data collection and the present publication of the findings. Furthermore, using both qualitative and quantitative research methods would have increased the generalisability of the findings on the framing of inclusive education in the teacher education programme. Finally, the limited time spent by the researcher in the field may have aided some fallacious claims by the participants.

## Footnotes

1 UNESCO’s (2014) roadmap for implementing the Global Action Programme on Education for Sustainable Development has five priority action areas: (1) advancing policy, (2) transforming learning and training environments, (3) building the capacities of educators and trainers, (4) empowering and mobilising youth and (5) accelerating sustainable solutions.

2 High numbers refer to a single class size of over 100 learners of diverse needs, manned by one teacher (in most cases).

3 Regular schools are education settings accessed by all of those of school-going age who do not need special accommodations.

4 Special needs education consists of education interventions and other supports designed to address special learning needs. Special education mainly comprises the education of children with disabilities, usually in special schools or institutions. The concept of children with special educational needs has been extended from students who have physical and other disabilities to include pupils who are failing in school for a variety of other reasons (Ministry of Education and Sports 2007).
